# Environmental Carcinogenesis as a Stochastic Evolutionary Failure of Senescence-Control Systems

**DOI:** 10.3390/cells15141234

**Published:** 2026-07-08

**Authors:** Jose-Ramon Blanco, Amancio Carnero

**Affiliations:** 1 Hospital Universitario San Pedro, 26006 Logroño, Spain; 2 Centro de Investigación Biomédica de La Rioja, 26006 Logroño, Spain; 3 Grupo de Investigación en Cuidados y Salud (GRUPAC), Universidad de La Rioja, 26004 Logroño, Spain; 4 Instituto de Biomedicina de Sevilla (IBIS), Hospital Universitario Virgen del Rocío (HUVR), Consejo Superior de Investigaciones Científicas (CSIC), Universidad de Sevilla, 41013 Sevilla, Spain; 5 CIBER de Cáncer (CIBERONC), Instituto de Salud Carlos III, 28029 Madrid, Spain

**Keywords:** cellular senescence, carcinogenesis, stochastic model, tumor initiation, environmental carcinogens, immortalization, mutagenesis, clonal evolution

## Abstract

**Highlights:**

**What are the main findings?**

- Environmental carcinogenesis is proposed as a probabilistic multistep process requiring both oncogenic mutation acquisition and escape from senescence-mediated growth arrest.
- Cellular senescence functions as a biological filter that limits clonal expansion, helping to explain the low efficiency of malignant transformation despite widespread carcinogen exposure.

**What is the implication of the main finding?**

- The study revises classical deterministic models of carcinogenesis by integrating stochastic cellular responses and senescence escape into tumor initiation.
- The model proposes that carcinogenic outcomes may exhibit nonlinear dose–response behavior, potentially reflecting the balance between mutation accumulation, cellular survival, and senescence-mediated growth suppression.

**Abstract:**

Environmental carcinogenesis is traditionally explained by the accumulation of genetic alterations induced by exogenous carcinogens. However, most exposed cells do not undergo malignant transformation because intrinsic tumor-suppressive mechanisms limit the expansion of damaged clones. Among these mechanisms, cellular senescence represents a major barrier that restricts proliferation following DNA damage, oncogenic stress, and other carcinogen-induced insults. In this review, we examine environmental carcinogenesis within a probabilistic evolutionary framework in which tumor initiation depends not only on mutation acquisition but also on the ability of rare cells to evade senescence-mediated growth arrest. Environmental carcinogens contribute to cancer development by increasing genomic instability, altering tissue microenvironments, and modifying selective pressures, whereas senescence acts as a critical constraint on clonal evolution. We further discuss how aging, immune surveillance, DNA-repair capacity, and tissue-specific factors influence the likelihood of senescence escape and malignant progression. This integrative perspective highlights carcinogenesis as a multistep stochastic process shaped by the interaction between mutational events, cellular fitness barriers, and microenvironmental selection. Understanding how these factors collectively regulate transformation may improve mechanistic models of cancer risk and identify new opportunities for prevention and early intervention.

## 1. Introduction

Environmental carcinogenesis has traditionally been understood as a consequence of cumulative genetic damage induced by exogenous agents. Classical multistep models propose that the progressive accumulation of oncogenic mutations eventually drives malignant transformation. Although this paradigm has provided a clear understanding of cancer development, it does not fully explain a fundamental biological observation: most cells exposed to carcinogens do not become malignant. Instead, the majority of damaged cells activate intrinsic tumor-suppressive responses that limit the propagation of genomic instability. Among these responses, cellular senescence represents one of the most important and evolutionarily conserved barriers to tumorigenesis. Cellular senescence is a stable, usually durable form of growth arrest that is triggered by diverse stresses, including DNA damage, oncogene activation, oxidative stress, mitochondrial dysfunction, and telomere erosion [1,2,3,4,5,6,7], and that can be bypassed under specific genetics or epigenetic conditions. Senescent cells remain metabolically active but lose proliferative capacity, thereby restricting the expansion of potentially malignant clones.

Senescence has traditionally been viewed as a protective mechanism that prevents malignant progression by arresting damaged cells. However, senescence also has broader biological implications. Senescent cells can influence tissue homeostasis through the senescence-associated secretory phenotype (SASP), which modulates inflammatory signaling, immune surveillance, extracellular matrix remodeling, and intercellular communication [1,2,3,4,5]. Consequently, senescence contributes not only to tumor suppression but also to the evolutionary dynamics of cell populations exposed to carcinogenic stress.

Although the accumulation of oncogenic mutations remains a central feature of carcinogenesis, the efficiency of malignant transformation is strongly influenced by biological barriers that limit the expansion of damaged cells. Among these barriers, cellular senescence plays a particularly important role by imposing stable growth arrest in response to genomic instability and other forms of cellular stress. Consequently, tumor initiation depends not only on the generation of oncogenic alterations but also on the ability of rare cells to overcome or bypass these protective mechanisms. In this context, carcinogenesis can be viewed as the outcome of interactions between mutation acquisition, cellular fitness constraints, and selective pressures operating within tissues over time.

Here, we propose that environmental carcinogenesis should be interpreted not only as a mutation-driven process, but as a probabilistic failure of senescence-mediated biological containment. This article therefore reframes environmental carcinogenesis, describing it as a stochastic, multistage evolutionary process shaped by senescence-dependent selection mechanisms. From this viewpoint, environmental carcinogens contribute mainly by elevating genomic instability and altering cellular selection dynamics, while senescence serves as a regulatory barrier that suppresses the expansion and persistence of potentially malignant cell populations.

## 2. Carcinogen-Induced Cellular Responses

Exposure to environmental carcinogens triggers a complex network of cellular stress responses that determine whether damaged cells survive, undergo repair, enter senescence, or are eliminated through apoptosis. The ultimate biological outcome depends on multiple factors, including the type of carcinogen, dose and duration of exposure, cellular context, tissue-specific characteristics, and the functional integrity of tumor-suppressive pathways.

Many environmental carcinogens, including polycyclic aromatic hydrocarbons, ultraviolet radiation, ionizing radiation, heavy metals, and tobacco-derived compounds, induce DNA lesions that activate the DNA damage response (DDR). This highly conserved signaling network is initiated through the activation of sensor proteins such as ATM (ataxia-telangiectasia mutated) and ATR (ATM and Rad3-related), which subsequently activate downstream checkpoint kinases, including CHK1 and CHK2. These signaling cascades stabilize p53 and induce transcriptional programs that regulate cell-cycle arrest, DNA repair, apoptosis, and senescence [6,7,8,9,10,11].

At relatively low levels of damage, efficient DNA-repair mechanisms may restore genomic integrity, allowing cells to resume proliferation. However, persistent DNA damage signaling can induce long-term growth arrest through activation of the p53/p21 and p16INK4A/pRB pathways, which represent the principal molecular regulators of cellular senescence [1,2,3,4,5,6,7]. The p53 pathway responds primarily to acute DNA damage and replication stress, whereas p16INK4A-mediated signaling contributes to the maintenance and stabilization of senescence. Together, these pathways prevent the propagation of cells harboring potentially oncogenic lesions.

In addition to DNA damage, carcinogens may induce oxidative stress, mitochondrial dysfunction, telomere erosion, and epigenetic alterations that further reinforce senescence programs. Chronic exposure to reactive oxygen species (ROS), for example, promotes genomic instability and can trigger premature senescence through activation of DDR signaling and inflammatory pathways [12,13,14,15,16,17]. Similarly, telomere shortening resulting from repeated cell division or genotoxic stress can induce replicative senescence, thereby limiting long-term clonal expansion [18,19].

Recent studies have also highlighted the importance of inflammatory signaling pathways in senescence induction. Persistent DNA damage can activate the cGAS-STING pathway through the accumulation of cytoplasmic chromatin fragments, leading to NF-κB activation and the development of the senescence-associated secretory phenotype (SASP). The SASP comprises cytokines, chemokines, growth factors, and matrix-remodeling enzymes that influence immune surveillance, tissue repair, and intercellular communication [1,2,3,4,5]. Although these responses initially contribute to tumor suppression by promoting immune-mediated clearance of damaged cells, persistent SASP signaling may also modify tissue microenvironments in ways that facilitate later stages of tumor evolution.

Importantly, carcinogen exposure does not produce uniform cellular outcomes. Even within genetically similar cell populations, substantial heterogeneity exists in damage recognition, DNA-repair capacity, metabolic activation of carcinogens, and stress-response signaling. Consequently, identical exposures may generate distinct outcomes, ranging from complete repair and recovery to apoptosis, senescence, or continued proliferation with acquired mutations. This heterogeneity is a critical determinant of transformation efficiency and contributes to the stochastic nature of carcinogenesis.

Experimental studies consistently demonstrate that immortalization and malignant transformation are rare outcomes following carcinogen exposure (Table 1). For example, chronic exposure of human mammary epithelial cells to benzo[a]pyrene generates only occasional immortalized clones despite widespread DNA damage [20]. Similarly, spontaneous immortalization frequencies in mouse embryonic fibroblasts typically occur at rates of approximately 10^−5^ to 10^−7^ cells and are frequently associated with disruption of the p53 or INK4A/ARF pathways [21,22]. Mutagenic stress may increase the frequency of such escape events, but carcinogens do not deterministically induce transformation. Instead, they increase the probability that rare cells acquire combinations of genetic and epigenetic alterations sufficient to overcome senescence-associated barriers.

Furthermore, efficient immortalization generally requires cooperative events affecting multiple regulatory pathways. Disruption of p53 and pRB signaling, activation of telomerase, acquisition of alternative telomere-maintenance mechanisms, and epigenetic silencing of tumor-suppressor genes often act together to permit sustained proliferation [22,23,24]. These observations indicate that carcinogenesis is not the inevitable consequence of mutagenesis but rather the outcome of rare evolutionary events occurring within highly constrained cellular populations.

Taken together, these findings support the view that environmental carcinogenesis is shaped not only by mutation generation but also by the balance between DNA repair, apoptosis, senescence induction, immune-mediated clearance, and the probability of escape from these protective barriers. The efficiency with which these responses operate ultimately determines whether carcinogen-exposed cells are eliminated, arrested, or permitted to participate in subsequent stages of tumor evolution.

These findings indicate that most damaged cells are either arrested or eliminated, raising the central question of how malignant transformation nonetheless emerges despite robust tumor-suppressive barriers.

### Relationship Between Mutation-Centered and Senescence-Dependent Models of Carcinogenesis

Classical models of carcinogenesis have traditionally emphasized the progressive accumulation of genetic and epigenetic alterations as the principal driving force underlying malignant transformation. Within this framework, environmental carcinogens contribute to cancer development primarily by increasing mutation rates, generating genomic instability, and promoting the acquisition of oncogenic alterations that confer selective growth advantages [7,9,20,21,22,23]. The multistage model of carcinogenesis, supported by extensive experimental and epidemiological evidence, remains fundamental to current understanding of cancer initiation and progression.

However, mutation accumulation alone does not fully explain the low efficiency of malignant transformation observed in most biological systems. Experimental studies consistently demonstrate that only a small fraction of carcinogen-exposed cells ultimately undergo immortalization or malignant conversion despite widespread DNA damage and mutagenesis [20,21,22,25,26]. This observation suggests that additional biological mechanisms influence the probability that mutated cells successfully contribute to tumor formation.

Cellular senescence represents one such mechanism. In response to DNA damage, oncogenic stress, telomere dysfunction, oxidative injury, or epigenetic perturbations, cells may undergo stable growth arrest through activation of tumor-suppressive pathways involving p53, p21, p16INK4A, and pRB [1,2,3,4,5,6,7]. These responses limit the proliferation of damaged cells and reduce the likelihood that oncogenic mutations become fixed within expanding cellular populations. Consequently, senescence acts as a major biological constraint on clonal evolution following carcinogenic exposure.

From this perspective, mutation-centered and senescence-dependent models should not be viewed as competing explanations but rather as complementary components of the same carcinogenic process. Mutation-centered models explain the generation of oncogenic diversity within tissues, whereas senescence-dependent models emphasize the selective barriers that determine whether mutated cells survive, persist, and expand. Tumor initiation therefore depends not only on the acquisition of oncogenic alterations but also on the probability that cells overcome growth-arrest mechanisms, evade immune surveillance, and adapt to tissue-specific selective pressures.

An important distinction between these perspectives concerns the interpretation of carcinogenic efficiency. In purely mutation-centered models, cancer risk is often assumed to increase as a function of accumulated genetic damage. In contrast, senescence-dependent frameworks propose that transformation probability reflects a balance between mutagenic processes and the effectiveness of tumor-suppressive mechanisms. Environmental carcinogens may increase mutation burden, but they simultaneously activate DNA-damage responses, apoptosis, immune surveillance, and senescence pathways that eliminate or restrain damaged cells [1,27,28]. As a result, carcinogenic outcomes emerge from the dynamic interaction between mutation generation and biological containment mechanisms.

This integrated view is particularly relevant in the context of aging. Age-related declines in DNA repair capacity, immune surveillance, and tissue homeostasis may weaken senescence-mediated constraints, thereby increasing the likelihood that mutated cells escape growth control and undergo malignant progression [28,29,30]. Thus, cancer risk reflects not only the cumulative burden of mutations acquired throughout life but also the progressive erosion of the systems that normally suppress clonal expansion.

Accordingly, the framework proposed in this review does not replace traditional multistage models of carcinogenesis. Rather, it extends them by incorporating senescence-mediated selection, tissue-level constraints, and aging-associated changes into a probabilistic and evolutionary interpretation of environmental carcinogenesis (Table 2). Under this view, malignant transformation is determined not simply by the generation of mutations but by the interplay between mutation acquisition, cellular fitness barriers, immune surveillance, and microenvironmental selection operating over time.

## 3. Senescence as a Population-Level Evolutionary Barrier

At the population level, senescence functions as a stringent evolutionary bottleneck that limits clonal expansion following carcinogenic exposure. Most damaged cells enter stable growth arrest, whereas only a small fraction retain proliferative capacity [3,21,26,27,31,32,33]. This asymmetry implies that carcinogenesis is fundamentally a Darwinian process of selection among rare surviving cells rather than a widespread or deterministic consequence of mutagenesis.

Thus, senescence acts not only as a tumor-suppressive mechanism but also as a major selective barrier at the tissue level, limiting the expansion of damaged cells and constraining the pool of clones available for further evolutionary selection. Cells capable of escaping senescence gain a substantial selective advantage because they retain the capacity for continued proliferation and additional mutation acquisition. Consequently, the emergence of malignant clones reflects both mutational processes and selective pressures imposed by senescence-mediated constraints.

Experimental evidence strongly supports this interpretation. Early studies demonstrated that immortalization precedes full malignant transformation and that cells must first bypass senescence before becoming susceptible to subsequent oncogenic progression [34,35,36,37]. Consistent with this idea, acquisition of limitless replicative potential remains a defining hallmark of cancer [28,29,38].

Importantly, mutation frequencies induced by environmental carcinogens, although elevated relative to baseline, remain relatively low in absolute terms (Table 3). This observation suggests that acquisition of the multiple alterations required for immortalization and transformation is intrinsically rare.

Even modest increases in mutation frequency may substantially elevate the probability of transformation when considered across large cell populations and repeated cell divisions. However, because multiple independent alterations are generally required to overcome senescence barriers, carcinogenesis remains a low-probability event. These considerations support a stochastic multistep model in which tumor initiation depends on the interplay between mutation supply, cellular survival, and selective escape from growth arrest.

## 4. Senescence-Associated Secretory Phenotype (SASP) and Tissue Microenvironmental Remodeling

While cellular senescence functions primarily as a tumor-suppressive mechanism through stable cell-cycle arrest, senescent cells remain metabolically active and profoundly influence their surrounding microenvironment through the senescence-associated secretory phenotype (SASP). The SASP consists of a complex network of cytokines, chemokines, growth factors, extracellular matrix-remodeling enzymes, and bioactive mediators that regulate intercellular communication, immune responses, tissue repair, and tissue remodeling [28,44]. Increasing evidence indicates that the biological consequences of senescence are determined not only by growth arrest itself but also by the composition, duration, and context of SASP signaling [28,45,46].

Early studies identified SASP components such as IL-6, IL-8, CCL2, VEGF, TGF-β, and matrix metalloproteinases as major mediators of senescence-associated inflammation [44]. However, recent transcriptomic, proteomic, and single-cell analyses have demonstrated that SASP composition is highly heterogeneous and varies according to cell type, senescence-inducing stimulus, tissue microenvironment, age, and duration of senescence [28,45,46]. Consequently, senescence should not be viewed as a single biological state but rather as a spectrum of related cellular programs capable of generating distinct secretory phenotypes and biological outcomes [47,48].

### 4.1. Heterogeneity of SASP Programs

Recent studies have revealed substantial heterogeneity among senescent cells. Different senescence-inducing stimuli, including DNA damage, oncogene activation, oxidative stress, mitochondrial dysfunction, telomere shortening, and epigenetic alterations, generate partially overlapping but distinct SASP profiles [28]. Furthermore, fibroblasts, epithelial cells, endothelial cells, and immune cells produce different combinations of inflammatory mediators despite sharing common senescence markers [28,45,46].

The growing recognition of senescence heterogeneity has important implications for carcinogenesis. Some SASP programs reinforce tumor suppression by facilitating immune-mediated clearance of damaged cells, whereas others may contribute to chronic inflammation, stem-cell dysfunction, extracellular matrix remodeling, and tumor promotion [28,45,46]. These observations help explain the apparently contradictory roles of senescence in cancer biology and support the view that the biological impact of senescent cells depends strongly on context [49,50].

### 4.2. Acute Versus Chronic Senescence

An important distinction emerging from recent work is the difference between acute and chronic senescence. Acute senescence is generally transient and contributes to physiological processes such as wound healing, embryonic development, tissue remodeling, and short-term responses to cellular injury [2,28]. In these contexts, SASP factors recruit immune cells that eliminate senescent cells and promote tissue restoration.

In contrast, chronic senescence is characterized by the long-term persistence of senescent cells and sustained SASP activity, frequently observed during aging and chronic environmental exposure [45,51,52,53]. Persistent senescent-cell accumulation may establish a state of low-grade chronic inflammation that contributes to tissue dysfunction and increased cancer susceptibility [28,45,52].

### 4.3. Immune Surveillance and Senescence

The interaction between senescence and the immune system represents a critical determinant of carcinogenic outcomes. SASP factors recruit natural killer cells, macrophages, neutrophils, dendritic cells, and T lymphocytes that participate in the recognition and elimination of damaged or premalignant cells [51,54]. This process, often referred to as senescence surveillance, reinforces the tumor-suppressive function of senescence by preventing the persistence of potentially malignant clones [28,35,51].

Recent evidence has highlighted the role of the cGAS-STING pathway in linking persistent DNA damage to inflammatory signaling. Cytoplasmic chromatin fragments generated during senescence activate cGAS-STING signaling, leading to NF-κB activation and amplification of SASP-associated inflammatory responses [1,28]. While these pathways contribute to immune-mediated clearance of damaged cells, persistent activation may also promote chronic inflammation when senescent cells accumulate over time.

Importantly, aging is associated with progressive impairment of immune surveillance mechanisms. Declining immune efficiency and immunosenescence reduce the clearance of senescent cells, thereby increasing tissue exposure to chronic SASP signaling. This phenomenon may contribute to the increased incidence of cancer observed in older individuals.

### 4.4. SASP and Tumor Promotion

Although senescence suppresses proliferation of damaged cells, persistent SASP activity may indirectly facilitate tumorigenesis. Long-term secretion of inflammatory cytokines, growth factors, and matrix-remodeling enzymes can alter tissue architecture, stimulate angiogenesis, enhance epithelial plasticity, and promote the survival and expansion of neighboring premalignant cells [44,54,55].

Experimental evidence indicates that senescent stromal cells can create tissue environments that favor tumor initiation and progression through paracrine signaling mechanisms [44,55]. Moreover, chronic inflammation associated with persistent senescent-cell accumulation has been linked to multiple hallmarks of cancer, including sustained proliferative signaling, resistance to cell death, angiogenesis, and altered immune responses [28,46,56].

Importantly, these protumorigenic effects do not imply that senescence itself is carcinogenic. Rather, they reflect the complex balance between the beneficial effects of growth arrest and the potentially detrimental consequences of prolonged inflammatory signaling [28,46]. Thus, the impact of senescence on carcinogenesis depends strongly on tissue context, duration of senescence, immune competence, and the efficiency of senescent-cell clearance mechanisms.

### 4.5. Implications for Environmental Carcinogenesis

Within the framework proposed here, environmental carcinogens influence cancer risk not only through direct mutagenesis but also through modulation of senescence-associated tissue environments. Carcinogen-induced senescence may initially suppress transformation by preventing the proliferation of damaged cells. However, chronic exposure can increase senescent-cell burden and sustain inflammatory SASP signaling, thereby modifying tissue architecture and altering selective pressures acting on surviving cell populations [9,27].

Consequently, environmental carcinogenesis should be considered a dynamic process involving interactions among mutation acquisition, senescence induction, immune surveillance, and microenvironmental remodeling. The balance among these factors ultimately determines whether carcinogen-exposed tissues successfully maintain homeostasis or progress toward malignant transformation [28,46].

## 5. Mechanisms of Senescence Escape and Malignant Progression

Although cellular senescence represents a major barrier to malignant transformation, senescence is not invariably irreversible. A small subset of damaged cells may acquire genetic, epigenetic, or adaptive alterations that permit escape from growth arrest and re-entry into the cell cycle. These rare events are of particular importance in carcinogenesis because they allow cells harboring genomic abnormalities to persist, proliferate, and accumulate additional oncogenic alterations. Consequently, malignant transformation often depends not only on mutation acquisition but also on the successful circumvention of senescence-control mechanisms.

### 5.1. Disruption of the p53 Pathway

The p53 signaling pathway is one of the principal mediators of senescence induction following DNA damage and oncogenic stress. Activation of p53 promotes transcription of p21CIP1, resulting in cell-cycle arrest and providing time for DNA repair or initiation of senescence programs [1,2,3,4,5,6,7]. Loss-of-function mutations in TP53, which occur in a large proportion of human cancers, impair these protective responses and allow damaged cells to continue proliferating despite persistent genomic instability [23,24].

Environmental carcinogens such as tobacco smoke, ultraviolet radiation, and polycyclic aromatic hydrocarbons frequently induce DNA lesions that target TP53 or its regulatory pathways. Cells carrying dysfunctional p53 are less likely to undergo senescence following carcinogenic exposure and therefore possess a greater probability of acquiring additional oncogenic alterations. Experimental studies have demonstrated that p53 inactivation substantially increases immortalization frequency and accelerates malignant progression in multiple cellular systems [21,22].

### 5.2. Inactivation of the pRb Pathway

The retinoblastoma protein (pRb) pathway represents a second major regulator of senescence. Activation of p16INK4A inhibits cyclin-dependent kinases 4 and 6, maintaining pRb in its active form and preventing cell-cycle progression through the G1/S checkpoint [1,2]. Persistent activation of the p16INK4A/pRb axis contributes to the maintenance and stabilization of senescence-associated growth arrest.

Genetic deletion, mutation, or epigenetic silencing of components of this pathway permits cells to bypass senescence and continue proliferating despite extensive cellular damage. In many cancers, simultaneous disruption of both p53 and pRb pathways is required for efficient immortalization, highlighting the cooperative nature of senescence-control mechanisms [22,26]. Loss of pRb-mediated regulation therefore represents a critical step in the transition from growth arrest to uncontrolled proliferation.

### 5.3. Telomerase Reactivation and Replicative Immortality

Replicative senescence is largely driven by progressive telomere shortening that occurs during repeated cell division. Critically short telomeres activate DNA damage responses and trigger growth arrest through p53-dependent and p53-independent mechanisms [18,19]. This process limits the proliferative lifespan of normal somatic cells and serves as an important barrier to tumorigenesis.

Many malignant cells overcome this limitation through reactivation of telomerase, a ribonucleoprotein complex that maintains telomere length and permits continued cellular replication [19,37,57]. Telomerase activation is observed in the majority of human cancers and is widely considered a hallmark of cellular immortalization. By preventing telomere-driven senescence, telomerase enables long-term clonal expansion and provides opportunities for continued mutation accumulation and evolutionary selection.

### 5.4. Alternative Lengthening of Telomeres (ALT)

Not all cancer cells rely on telomerase for telomere maintenance. A subset of tumors utilizes alternative lengthening of telomeres (ALT), a recombination-based mechanism that preserves telomere integrity independently of telomerase activity [18,19]. ALT is particularly common in certain sarcomas, gliomas, and other mesenchymal malignancies.

The acquisition of ALT provides an alternative route to senescence escape by preventing telomere dysfunction and extending replicative lifespan. Although less common than telomerase activation, ALT illustrates the existence of multiple evolutionary solutions through which damaged cells can overcome proliferative barriers and achieve immortality.

### 5.5. Oncogene-Induced Senescence Bypass

Activation of oncogenes such as RAS, BRAF, MYC, and others can paradoxically induce senescence rather than transformation, a phenomenon known as oncogene-induced senescence (OIS) [3,32,33]. OIS serves as a protective response that prevents aberrant proliferation following excessive mitogenic signaling.

However, additional genetic alterations affecting p53, p16INK4A, pRb, DNA damage responses, or chromatin regulators may permit cells to bypass OIS. Once these barriers are removed, oncogenic signaling can drive sustained proliferation, genomic instability, and clonal expansion. Experimental and clinical studies suggest that escape from OIS frequently represents an early step in tumor initiation, particularly in epithelial malignancies [3,32,33].

### 5.6. Epigenetic Reprogramming and Senescence Escape

Increasing evidence indicates that epigenetic alterations play important roles in regulating senescence stability. Changes in DNA methylation, histone modifications, chromatin organization, and non-coding RNA expression can influence the expression of key senescence regulators, including TP53, CDKN2A, RB1, and numerous SASP-associated genes [4,58,59].

Environmental carcinogens may promote epigenetic remodeling either directly or indirectly through chronic inflammation, oxidative stress, and DNA damage. Such alterations can weaken senescence programs without requiring structural mutations in canonical tumor-suppressor genes. Epigenetic reprogramming may therefore provide an additional mechanism through which damaged cells escape growth arrest and acquire increased malignant potential.

### 5.7. Senescence Escape as a Rare Evolutionary Event

Importantly, senescence escape remains a relatively uncommon outcome compared with repair, apoptosis, or stable growth arrest. Most carcinogen-exposed cells never acquire the combination of molecular alterations necessary to overcome multiple senescence-control systems. Nevertheless, because escaped cells retain proliferative capacity while harboring accumulated genetic and epigenetic abnormalities, they possess a substantial selective advantage within evolving tissue populations.

Within the stochastic framework proposed in this review, malignant transformation can therefore be viewed as the consequence of rare evolutionary events in which damaged cells successfully evade senescence, survive tissue-level selection pressures, and undergo progressive clonal expansion. The probability of transformation depends not only on mutation generation but also on the efficiency of the biological barriers that normally prevent senescence escape.

## 6. Dose as a Determinant of Carcinogenic Outcome

The probability of malignant transformation is determined not only by mutation acquisition but also by the capacity of cells to survive carcinogenic exposure. Increasing carcinogen dose may simultaneously enhance mutagenesis while also increasing cytotoxicity, apoptosis, and senescence induction. Consequently, the relationship between exposure intensity and transformation probability may not necessarily be linear.

Within the stochastic framework proposed in this review, we hypothesize that transformation risk may emerge from the balance between two opposing processes: (i) the generation of genetic and epigenetic alterations that increase the likelihood of senescence escape and (ii) the elimination or permanent growth arrest of damaged cells through apoptosis and senescence. At very low exposure levels, the frequency of oncogenic alterations may be insufficient to support the accumulation of the multiple events required for malignant progression. At very high exposure levels, extensive cytotoxicity and persistent activation of tumor-suppressive pathways may reduce the survival of potentially transformable cells. Between these extremes, intermediate exposure conditions could theoretically provide a permissive window in which mutation accumulation and cellular survival coexist.

Importantly, this proposed relationship should be regarded as a conceptual hypothesis rather than an established empirical dose–response model. The present review does not propose a universal bell-shaped relationship between carcinogen exposure and cancer risk. Rather, this hypothesis illustrates how mutagenesis, cellular viability and senescence-mediated selection may interact to produce context-dependent nonlinear outcomes. under certain biological conditions. Direct experimental support for such a transformation window remains limited; its validation will require dose-resolved systems capable of simultaneously quantifying mutation acquisition, senescence induction, cellular survival, and clonal expansion.

Nonlinear dose–response relationships have been recognized in toxicology and risk assessment, where biological responses cannot always be adequately described by simple linear models across all exposure ranges [60,61,62,63,64]. Likewise, hormetic responses, characterized by qualitatively different biological effects at low and high doses, have been reported for diverse chemical and physical stressors, including radiation, oxidative stress, and environmental toxicants [62,63,64,65,66,67]. Although the relevance of hormesis to carcinogenesis remains debated and likely depends on biological context, these observations support the broader principle that biological responses to environmental exposures may reflect the interaction of adaptive, protective, and damaging mechanisms rather than dose alone [62,63,64,65,66,67].

Accordingly, the framework proposed here predicts only that a carcinogenic outcome may depend on the dynamic balance among mutation generation, cellular survival, senescence induction, immune surveillance, and tissue-specific selection pressures. Future experimental studies using dose-resolved carcinogen exposure systems, lineage-tracing approaches, and single-cell analyses will be required to determine whether such nonlinear transformation dynamics occur in specific biological contexts and to define the conditions under which they may be observed.

## 7. Aging as a Modulator of Senescence-Dependent Carcinogenesis

Age is one of the strongest risk factors for cancer development, yet the mechanisms underlying this association extend beyond the simple accumulation of mutations over time. Aging affects multiple biological processes that influence the probability that carcinogen-exposed cells survive, evade senescence, and ultimately acquire malignant potential. Consequently, the relationship between aging and carcinogenesis is best understood as the progressive decline of cellular and tissue-level mechanisms that normally preserve multicellular homeostasis [49,50,68].

### 7.1. Age-Associated Decline in DNA Repair Capacity

The maintenance of genomic integrity depends on several DNA repair pathways, including nucleotide excision repair (NER), base excision repair (BER), homologous recombination (HR), and non-homologous end joining (NHEJ). These systems detect and repair DNA lesions generated by endogenous metabolic processes and environmental carcinogens [1,69,70].

Numerous studies have demonstrated that DNA repair efficiency declines with age, resulting in increased persistence of DNA damage and greater genomic instability [29,56,69]. Reduced repair capacity may increase the probability that carcinogen-induced lesions become permanently fixed as mutations during cell division. Furthermore, chronic DNA damage signaling contributes to senescence induction, inflammatory responses, and tissue dysfunction, thereby altering the microenvironment in which carcinogenesis occurs [12,14,42].

Importantly, aging does not simply increase mutation burden. It also affects the ability of tissues to eliminate or contain damaged cells. Thus, carcinogenic risk reflects the combined effects of mutation accumulation and declining protective mechanisms.

### 7.2. Immunosenescence and Declining Senescence Surveillance

The immune system plays a critical role in identifying and eliminating damaged, senescent, and premalignant cells. Senescence-associated secretory phenotype (SASP) factors recruit natural killer cells, macrophages, dendritic cells, and T lymphocytes that contribute to senescent-cell clearance and tumor suppression [51,54].

With aging, however, immune function undergoes progressive deterioration, a process commonly referred to as immunosenescence. Age-related reductions in immune surveillance impair the clearance of damaged and senescent cells, allowing these populations to accumulate within tissues [30,51]. Persistent senescent-cell accumulation may increase exposure to chronic SASP signaling, thereby modifying tissue architecture, promoting inflammatory remodeling, and altering selective pressures acting on neighboring cell populations [28,45,46].

As a result, aged tissues may exhibit both increased mutation burden and reduced capacity to remove potentially dangerous cellular clones, increasing the probability of malignant progression.

### 7.3. Inflammaging and Tissue Remodeling

Aging is also associated with the development of chronic low-grade inflammation, commonly termed inflammaging. This condition is characterized by persistent elevation of inflammatory mediators, including IL-6, TNF-α, and other cytokines associated with SASP signaling [30].

The accumulation of senescent cells represents a major contributor to inflammaging. Chronic inflammatory signaling can disrupt tissue organization, impair stem-cell function, alter extracellular matrix composition, and promote cellular plasticity [28,30,45,46]. These changes may create tissue environments that favor the survival and expansion of premalignant clones. Within this framework, inflammaging may be viewed as a progressive alteration of tissue-level selective landscapes. By modifying local ecological conditions, chronic inflammation can influence which cellular populations survive, compete, and expand following carcinogenic exposure.

### 7.4. Aging and Evolutionary Constraints on Carcinogenesis

The incidence of cancer increases dramatically with age despite the existence of multiple tumor-suppressive mechanisms. This apparent paradox reflects the gradual erosion of biological systems that normally constrain clonal evolution. DNA repair, immune surveillance, stem-cell regulation, and senescence-mediated growth arrest collectively function as barriers to malignant transformation throughout life [1,28,30].

As these protective systems decline, the probability that carcinogen-exposed cells evade growth control mechanisms increases. Therefore, aging can be viewed not simply as a period of cumulative damage accumulation but also as a progressive weakening of the evolutionary constraints that suppress malignant evolution.

## 8. Tissue-Specific Determinants of Carcinogenic Outcomes

Although environmental carcinogens may induce similar types of molecular damage, cancer incidence varies markedly among tissues. This observation suggests that tissue-specific factors strongly influence transformation efficiency and determine how senescence-mediated barriers operate in different biological contexts.

### 8.1. Regenerative Capacity and Cellular Turnover

Tissues differ substantially in their rates of cellular turnover and regenerative activity. Highly proliferative tissues, such as the intestinal epithelium and epidermis, continuously replace damaged cells and may therefore tolerate higher levels of environmental exposure while maintaining tissue integrity. In contrast, tissues with limited regenerative potential may accumulate damaged or senescent cells over time [29,30].

The probability that carcinogen-induced mutations become fixed within cellular populations depends partly on these differences in turnover dynamics. Tissues with active stem-cell compartments may provide greater opportunities for clonal selection and expansion following mutagenic exposure.

### 8.2. Tissue-Specific Determinants of Senescence-Dependent Carcinogenesis

Although environmental carcinogens often induce similar forms of DNA damage, cancer incidence and transformation efficiency vary substantially among tissues. These differences suggest that tissue-specific biological characteristics influence the balance between mutation accumulation, senescence induction, immune surveillance, and clonal expansion. Factors such as regenerative capacity, stem-cell dynamics, metabolic activity, local immune composition, and baseline senescent-cell burden can all affect carcinogenic outcomes (Table 4).

Lung

The lung is continuously exposed to inhaled environmental carcinogens, including tobacco smoke, air pollutants, and occupational toxicants. These exposures generate DNA adducts, oxidative stress, and chronic inflammation within airway epithelial cells [17,29]. Cellular senescence serves as an important protective mechanism by limiting the proliferation of damaged cells following genotoxic injury. However, persistent exposure can lead to the accumulation of senescent cells and chronic SASP-mediated inflammation, contributing to tissue remodeling and altered cellular selection pressures. The combination of repeated injury, inflammatory signaling, and progressive genomic instability may increase the likelihood of malignant transformation in susceptible cell populations.

Skin

The skin provides a well-characterized example of environmental carcinogenesis driven by ultraviolet (UV) radiation. UV exposure induces DNA photoproducts, oxidative damage, and activation of DNA damage response pathways [14,34]. Most damaged keratinocytes undergo DNA repair, apoptosis, or senescence, thereby preventing propagation of potentially oncogenic mutations. Nevertheless, occasional cells may evade these protective responses and accumulate additional alterations that contribute to the development of cutaneous malignancies. The high regenerative capacity of the epidermis allows efficient removal of many damaged cells, but repeated UV exposure may eventually overwhelm these protective mechanisms.

Liver

The liver is exposed to a diverse range of environmental and metabolic carcinogens, including aflatoxins, alcohol-related metabolites, and industrial toxicants. Hepatic tissues possess substantial regenerative capacity, which facilitates recovery following injury but also creates opportunities for clonal expansion of damaged cells [68]. Chronic exposure to hepatotoxic agents often results in persistent inflammation, fibrosis, and senescence accumulation. In this setting, SASP-mediated signaling may alter tissue architecture and promote a microenvironment favorable for hepatocellular carcinoma development. The interplay among regeneration, inflammation, and senescence therefore plays a particularly important role in liver carcinogenesis.

Colon

The intestinal epithelium undergoes continuous renewal throughout life and contains highly active stem-cell compartments that maintain tissue integrity. Environmental exposures, dietary factors, microbiome-derived metabolites, and chronic inflammatory conditions can influence mutation rates and selective pressures within these stem-cell populations [56]. Senescence limits the expansion of damaged cells, but alterations affecting growth-arrest pathways may allow mutated clones to persist and compete for niche occupancy. Because of the high turnover rate of the colonic epithelium, carcinogenic outcomes are strongly influenced by stem-cell dynamics, tissue organization, and local immune responses.

Implications for the Proposed Framework

These examples illustrate that environmental carcinogenesis cannot be fully understood through mutation burden alone (Table 4). The probability that carcinogen-induced mutations result in malignant transformation depends on tissue-specific characteristics that regulate cellular survival, senescence induction, immune-mediated clearance, and clonal competition. Consequently, the same environmental exposure may produce markedly different biological outcomes depending on the tissue involved. Incorporating these tissue-level determinants into models of carcinogenesis may improve our understanding of cancer susceptibility and help explain organ-specific differences in cancer incidence.

### 8.3. Tissue Ecology and Clonal Competition

Recent studies increasingly support the concept that tissues function as ecological systems in which cellular populations compete for space, nutrients, and growth signals. Within this framework, senescence serves as an important regulator of cellular fitness by removing or arresting damaged competitors.

Environmental carcinogens alter these ecological interactions by modifying mutation rates, inflammatory signaling, and tissue architecture. Consequently, malignant transformation depends not only on the acquisition of oncogenic mutations but also on the ability of emerging clones to compete successfully within changing tissue environments.

### 8.4. Implications for the Stochastic Model

The substantial variation in cancer incidence among tissues highlights the importance of tissue-specific selective pressures in carcinogenesis. Differences in DNA repair capacity, immune composition, regenerative potential, metabolic activity, and baseline senescent-cell burden can all influence transformation probability [28,29,45].

Therefore, environmental carcinogenesis should not be considered a uniform process across organs. Rather, carcinogenic outcomes emerge from interactions among mutagenic exposure, tissue-specific biology, senescence-mediated constraints, and age-related changes in cellular fitness landscapes.

## 9. An Integrative Stochastic Model of Carcinogenesis

**Figure 1:**
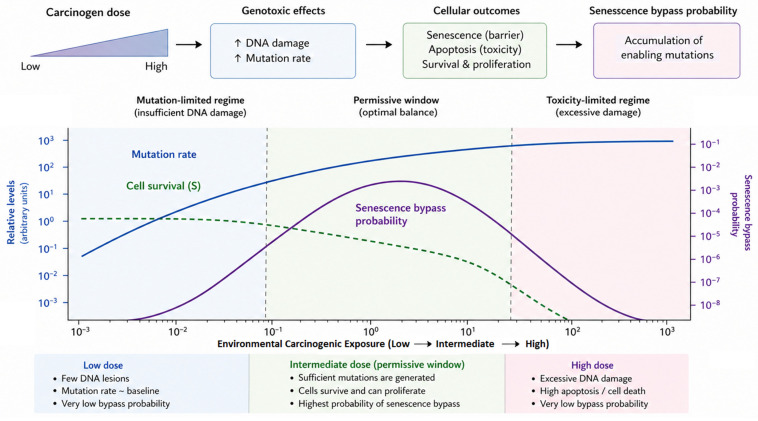
Conceptual model of senescence-dependent environmental carcinogenesis.

The considerations discussed above support a conceptual model in which environmental carcinogenesis emerges from the interaction between mutation accumulation, cell survival, and senescence escape. Within this model, senescence functions as a probabilistic barrier that constrains the evolutionary progression of damaged cell populations. Carcinogens increase mutation rates and selective pressures, thereby expanding the pool of genetically altered cells. However, only exceptionally rare cells acquire combinations of genetic and epigenetic alterations sufficient to bypass senescence while simultaneously maintaining viability and proliferative capacity. The conceptual relationship between carcinogen exposure, senescence induction, and transformation probability is summarized in Figure 1.

This model therefore differs fundamentally from deterministic models in which mutation accumulation alone drives malignant transformation. Instead, carcinogenesis is understood as a stochastic evolutionary process governed by low-probability escape events occurring within highly constrained cellular populations. The novelty of this review lies in synthesizing these observations into an integrated probabilistic framework linking carcinogenesis, senescence, aging, and evolutionary selection.

This framework generates several experimentally testable predictions:

1. Most carcinogen-exposed cells will undergo senescence or apoptosis rather than transformation.
2. Immortalization represents a critical intermediate step preceding malignant progression.
3. Transformation probability may exhibit nonlinear dose–response behavior depending on the balance among mutagenesis, cellular survival, and senescence-mediated constraints.
4. Disruption of senescence pathways may enhance carcinogenic risk independently of direct mutagenic activity.
5. Tissue microenvironmental factors and immune surveillance influence the selection of rare escape clones.

(1) Low levels of carcinogen exposure induce limited DNA damage that is efficiently repaired, resulting in minimal long-term biological consequences. (2) Intermediate levels of exposure increase mutation acquisition while maintaining sufficient cellular viability to permit clonal evolution, creating conditions that may favor malignant transformation. (3) Senescence, apoptosis, immune surveillance, and DNA repair mechanisms act as protective barriers that restrict the expansion of damaged cell populations. (4) At high levels of carcinogen exposure, extensive cytotoxicity, persistent DNA damage responses, and widespread senescence reduce cellular survival and limit the emergence of transformed clones.

The x-axis represents a conceptual gradient of carcinogen exposure intensity and does not correspond to a specific quantitative scale. The schematic integrates established concepts from multistage carcinogenesis, cellular senescence, DNA damage responses, and evolutionary models of tumor development. The figure is intended as a conceptual framework rather than a quantitative prediction of cancer risk. Based on concepts described in recent reviews of senescence-associated carcinogenesis such as [1,2,3,4,5,6,7,30,49,50,56,71].

As illustrated in Figure 1, malignant transformation is expected to depend on the balance between mutagenesis and senescence-mediated growth suppression.

## 10. Implications for Cancer Biology and Risk Assessment

This probabilistic model has several important implications for understanding environmental carcinogenesis and cancer risk.

First, it suggests that carcinogenic potential cannot be inferred solely from mutagenic activity. Risk assessment must additionally consider factors that influence senescence induction, cellular survival, immune clearance, and the probability of escape from growth arrest. Agents that impair senescence pathways may therefore enhance carcinogenic susceptibility even in the absence of strong direct mutagenic effects [9,27,72].

Second, this perspective emphasizes the importance of combinatorial and co-exposure effects. Environmental mixtures may alter carcinogenic outcomes not only by increasing mutational burden but also by modifying cellular stress responses, inflammatory signaling, or tissue microenvironments that regulate senescence and clonal selection.

Third, the proposed model strengthens conceptual links between carcinogenesis and aging biology. Aging tissues accumulate senescent cells, genomic instability, epigenetic alterations, and chronic inflammatory signals that may collectively modify the probability landscape governing transformation. In this sense, aging itself may represent the cumulative outcome of stochastic cellular events shaped by the dynamic balance between senescence, survival, and clonal evolution.

Finally, integrating senescence biology into models of environmental carcinogenesis may provide new opportunities for prevention and therapeutic intervention. Strategies that reinforce senescence barriers, improve immune-mediated clearance of damaged cells, or prevent senescence escape could potentially reduce the emergence of malignant clones during early carcinogenesis. A key future change will be to quantify these probabilities experimentally using longitudinal single-cell lineage tracing, senescence reporters, and dose-resolved carcinogen-exposure models.

## 11. Future Directions and Therapeutic Implications

The stochastic model proposed here provides a conceptual framework that may help integrate senescence biology into future approaches for cancer prevention and therapy. If senescence functions as a probabilistic barrier to carcinogenesis, therapeutic strategies capable of reinforcing this barrier or preventing senescence escape could potentially reduce tumor initiation.

One important implication concerns the development of senescence-modulating therapies. Pharmacological approaches that enhance p53 signaling, preserve pRb pathway integrity, or stabilize telomere-associated checkpoints may strengthen cellular resistance to malignant transformation [9,10,22,23,24]. Conversely, therapies aimed at selectively eliminating persistent senescent cells (senolytic strategies) may reduce chronic inflammatory signaling and limit pro-tumorigenic microenvironmental effects associated with long-term SASP activity [73,74,75,76,77]. However, these senolytic interventions may carry potential limitations and risks.

The elimination of senescent cells through senolytic therapies has emerged as a promising strategy to reduce chronic senescence-associated secretory phenotype (SASP) signaling and alleviate age-related tissue dysfunction. Experimental studies have demonstrated that senolytic agents can decrease senescent-cell burden, reduce chronic inflammation, improve tissue function, and, in some contexts, suppress tumor-promoting microenvironmental effects associated with persistent SASP activity [3,4,51,57,58,70].

However, the potential benefits of senolytic therapies must be considered in light of the physiological functions of cellular senescence. Senescence evolved as a protective mechanism that limits the proliferation of damaged cells, suppresses malignant transformation, contributes to tissue remodeling, and facilitates wound healing and tissue repair [1,28]. In addition, senescent cells can promote immune-mediated clearance of damaged cellular populations through SASP-dependent recruitment of immune effectors [1,3,51]. Consequently, indiscriminate elimination of senescent cells may have unintended biological consequences.

One important consideration is timing. Transient senescence induced during acute tissue injury often contributes to regeneration and restoration of tissue homeostasis. Premature removal of these senescent cells could impair wound healing, tissue remodeling, or normal repair processes [28,45]. In contrast, chronic accumulation of senescent cells during aging or prolonged environmental exposure may promote persistent inflammation and create microenvironments that favor tumor progression. Thus, the effects of senolytic intervention may depend critically on whether senescence is acute and beneficial or chronic and maladaptive.

Tissue-specific context also represents an important determinant of therapeutic outcome. Different tissues exhibit distinct rates of senescent-cell accumulation, regenerative capacity, immune composition, and susceptibility to carcinogenic processes. As a result, senolytic interventions that are beneficial in one biological setting may not produce equivalent effects in another. Furthermore, because senescent cells are heterogeneous and exhibit diverse secretory phenotypes, broad senolytic approaches may eliminate both harmful and potentially beneficial senescent-cell populations [28,46].

Additional concerns include incomplete target specificity, potential off-target effects, and uncertainty regarding the long-term consequences of repeated senolytic treatment. The selective removal of senescent cells may alter tissue homeostasis, stem-cell dynamics, and immune responses in ways that remain incompletely understood. Therefore, further investigation is required to determine the optimal timing, dosing strategies, and patient populations most likely to benefit from senescence-targeted interventions.

Within the framework proposed in this review, senolytic therapies should not be viewed as universally protective against carcinogenesis. Rather, their effects are likely to depend on the balance between reducing chronic SASP-mediated protumorigenic signaling and preserving the beneficial tumor-suppressive, regenerative, and homeostatic functions of senescence. Future therapeutic approaches may therefore benefit from selectively modulating harmful senescence-associated pathways while maintaining the protective aspects of senescence biology.

The proposed model also highlights the importance of studying non-mutagenic carcinogenic mechanisms. Environmental agents that interfere with senescence pathways, immune surveillance, or tissue homeostasis may significantly influence transformation probability even when direct mutagenicity is limited [8,24]. Future toxicological assessment models may therefore benefit from incorporating biomarkers related not only to DNA damage but also to senescence induction, inflammatory signaling, and immune-mediated clearance.

Additionally, integration of stochastic and evolutionary concepts into carcinogenesis research may improve understanding of interindividual variability in cancer susceptibility. Genetic background, aging, metabolic state, tissue regenerative capacity, and environmental co-exposures likely influence the probability of senescence escape in complex and dynamic ways. Systems-biology and computational approaches integrating these variables could help generate more biologically realistic predictive models of carcinogenic risk.

Recent advances in artificial intelligence (AI), machine learning (ML), systems biology, and computational oncology provide new opportunities to investigate the complex and stochastic nature of environmental carcinogenesis (see, for example, [78]). Traditional experimental approaches often capture only isolated components of tumor initiation, whereas carcinogenesis emerges from dynamic interactions among mutational processes, DNA repair mechanisms, cellular senescence, immune surveillance, tissue microenvironments, and aging-related changes. Computational models offer the possibility of integrating these diverse biological variables into unified predictive frameworks.

Machine-learning approaches applied to genomic, transcriptomic, epigenomic, proteomic, and single-cell datasets may help identify molecular signatures associated with senescence induction, persistence, and escape. Such models could improve the prediction of which carcinogen-exposed cell populations are most likely to bypass senescence-mediated growth arrest and acquire malignant potential. Similarly, AI-based analyses of large epidemiological and exposome datasets may facilitate the identification of environmental exposure patterns associated with increased carcinogenic risk.

Systems biology and agent-based modeling approaches may also provide valuable tools for investigating how individual cellular behaviors collectively generate tissue-level carcinogenic outcomes. By incorporating mutation acquisition rates, DNA repair efficiency, senescence dynamics, immune-cell interactions, and age-associated physiological changes, these models could help quantify the probability of transformation under different environmental conditions. In particular, computational simulations may be useful for evaluating the hypothesis proposed in this review that carcinogenesis reflects a probabilistic failure of senescence-control systems rather than a purely deterministic consequence of mutation accumulation.

Future integration of multi-omic datasets, longitudinal clinical data, and environmental exposure information through AI-driven platforms may ultimately improve cancer risk stratification, identify biomarkers of senescence escape, and support the development of personalized prevention strategies. As increasingly comprehensive biological datasets become available, computational approaches are likely to play a central role in understanding how environmental carcinogens, aging, and senescence interact to shape cancer susceptibility.

Finally, this perspective reinforces the need to study early carcinogenesis as a dynamic evolutionary process rather than a purely mutation-driven event. Understanding how rare damaged cells survive, evade senescence, and expand within tissue ecosystems may reveal novel opportunities for early intervention before irreversible malignant progression occurs.

## 12. Conclusions

Cellular senescence should be considered not merely a passive tumor-suppressive mechanism but an active probabilistic barrier that determines whether carcinogen-exposed cells progress toward malignancy. Environmental carcinogens do not directly or uniformly induce cancer; rather, they increase the likelihood that rare cells acquire combinations of alterations enabling survival, senescence bypass, and sustained proliferation.

By integrating mutation dynamics with senescence-mediated selection, the stochastic framework proposed here provides a biologically realistic explanation for the low efficiency of malignant transformation despite extensive carcinogen exposure. This perspective further predicts nonlinear dose–response relationships and highlights the importance of cellular context, tissue environment, and evolutionary selection in tumor initiation.

In this model, carcinogenesis is not interpreted as the deterministic consequence of mutational accumulation, but as a rare evolutionary outcome arising when multiple layers of biological fidelity control progressively fail. DNA repair limits mutation accumulation, senescence restricts propagation of damaged clones, apoptosis removes irreversibly compromised cells, and immune surveillance constrains clonal persistence. Aging gradually destabilizes these integrated barriers, thereby reshaping the probabilistic landscape that governs transformation. Cancer emergence therefore reflects a stochastic failure of multicellular homeostasis occurring across time, selection pressure, and tissue evolution.

## Figures and Tables

**Table 1:** Representative experimental evidence linking carcinogen exposure, senescence bypass, and immortalization.

Carcinogen or Stimulus	Experimental Model	Principal Cellular Response	Transformation Outcome	Key Implication
Benzo[a]pyrene	Human mammary epithelial cells	DNA damage and replication stress	Rare immortalized clones	Carcinogen exposure alone is insufficient for deterministic transformation
Ionizing radiation	Fibroblasts and MEFs	DNA double-strand breaks, senescence, apoptosis	Increased escape frequency	Mutagenesis increases the probability of senescence bypass
Oxidative stress	Multiple mammalian cell types	Stress-induced premature senescence	Genomic instability under chronic exposure	Persistent low-level stress may facilitate clonal selection
HPV E6/E7 expression	Human epithelial cells	p53 and pRb pathway inhibition	Efficient immortalization	Direct disruption of senescence pathways strongly enhances escape
Telomere dysfunction	Human primary cells	Replicative senescence and crisis	Rare immortalization after telomerase activation	Replicative barriers constrain long-term clonal expansion

**Table 2:** Summary table with explicit comparisons of the models.

Feature	Classical Mutation-Centered Model	Senescence-Dependent Framework
Primary focus	Mutation accumulation	Mutation accumulation + selective barriers
Role of carcinogens	Increase mutation burden	Increase mutation burden and activate senescence
Transformation efficiency	Mainly mutation-driven	Determined by mutation plus escape from constraints
Key determinants	Oncogenic mutations	Mutations, senescence, immune surveillance, tissue context
Aging effect	Accumulation of mutations	Mutations plus decline of protective mechanisms
Interpretation of cancer risk	Function of genetic damage	Function of genetic damage and failure of containment systems

**Table 3:** Representative examples of carcinogen-induced mutagenesis, senescence, and transformation outcomes in experimental systems.

Carcinogen/Exposure	Experimental System	Biological Outcome	Reported Mutation Frequency or Transformation Efficiency *	Senescence Involvement	Key Reference
Benzo[a]pyrene (B[a]P)	Human mammary epithelial cells (HMECs)	DNA adduct formation, immortalization of rare clones	Immortalization observed only in rare surviving clones	Senescence limits expansion of damaged cells	Stampfer & Bartley, 1985 [20]
Benzo[a]pyrene diol epoxide (BPDE)	Human bronchial epithelial cells	Extensive DNA damage and mutagenesis	Elevated DNA adduct burden; transformation remains infrequent	Persistent damage induces senescence and growth arrest	Rojas et al., 2004 [39]
Diesel exhaust carcinogens	Hupki mouse embryo fibroblasts (human TP53 knock-in)	TP53 mutations and genomic instability	Mutation induction dependent on metabolic activation	Senescence restricts expansion of damaged cells	Kucab et al., 2012 [40]
Chemical carcinogens	Syrian hamster embryo (SHE) cells	Senescence bypass and cellular transformation	Transformation frequency typically <10^−4^–10^−5^ surviving cells	Senescence bypass required for immortalization	Pickles et al., 2016 [25]
Carcinogen exposure (multiple agents)	Normal diploid mammalian cells	Senescence escape through p53/p16 alterations	Rare immortalization events (~10^−5^–10^−7^ cells)	Loss of senescence pathways required	Yasaei et al., 2013 [26]
Spontaneous or stress-induced mutagenesis	Mouse embryonic fibroblasts (MEFs)	Immortalization and genomic instability	Immortalization frequency approximately 10^−5^–10^−7^ cells	Senescence acts as major proliferative barrier	vom Brocke et al., 2006 [21]
Tobacco-smoke related carcinogens	Human bronchial epithelial cells	DNA damage, oxidative stress, mutation accumulation	Mutation burden increased but transformation remains inefficient	Senescence and apoptosis eliminate most damaged cells	Shah et al., 2016 [41]
Ionizing radiation	Bone marrow mesenchymal stem/stromal cells	DNA damage, ROS generation, senescence	Dose-dependent mutation induction; transformation rare	Senescence is a dominant response to persistent DNA damage	Chen et al., 2019 [14]; Li et al., 2026 [42]
Ionizing radiation	Rodent aging models	Genotoxic stress and age-related susceptibility	Increased mutation burden with age	Senescence contributes to tissue resilience against transformation	Csiszar et al., 2019 [43]

* Reported values are approximate and derived from the original studies. Transformation and immortalization frequencies vary considerably according to exposure conditions, cell type, and experimental design.

**Table 4:** Tissue-Specific Examples of Environmental Carcinogenesis.

Tissue	Major Environmental Carcinogens	Dominant Protective Mechanisms	Key Factors Affecting Transformation
Lung	Tobacco smoke, air pollution	Senescence, immune surveillance	Chronic inflammation, oxidative stress
Skin	UV radiation	DNA repair, apoptosis, senescence	Repeated exposure, stem-cell survival
Liver	Aflatoxins, alcohol metabolites	Regeneration, senescence	Chronic inflammation, fibrosis
Colon	Dietary carcinogens, inflammation	Stem-cell turnover, senescence	Niche competition, microbiome effects

## Data Availability

No new data were created or analyzed in this study.
